# Microbiome affects mice metabolic homeostasis via differential regulation of gene expression in the brain and gut

**DOI:** 10.14814/phy2.70373

**Published:** 2025-05-19

**Authors:** Wynne Milhouse, Anna Clapp Organski, Xun Sun, Derek Ai, Baohua Zhou, Tzu‐Wen L. Cross, Hongxia Ren

**Affiliations:** ^1^ Department of Pediatrics Herman B. Wells Center for Pediatric Research, Indiana University School of Medicine Indianapolis Indiana USA; ^2^ Center for Diabetes and Metabolic Disease Indiana University School of Medicine Indianapolis Indiana USA; ^3^ Stark Neurosciences Research Institute, Indiana University School of Medicine Indianapolis Indiana USA; ^4^ Department of Nutrition Science Purdue University West Lafayette Indiana USA

**Keywords:** brain‐gut axis, diabetes, endocrine, GI, GPCR, integrative, leptin, metabolism, microbiome, nutrient, obesity, sex

## Abstract

The gut microbiome (GMB) regulates digestion, metabolism, immunity, and energy homeostasis. This study investigates how gut microbiota integrate the regulation in the neuroendocrine and enteroendocrine systems, with a focus on G protein‐coupled receptors (GPCRs) in the brain‐gut axis and sex differences. Germ‐free (GF) mice exhibited increased hypothalamic expression of the anorexigenic neuropeptide and decreased expression of the negative regulator of leptin signaling. GF males had significantly lower serum leptin levels compared to conventional (CON) males, highlighting a potential link between the microbiome and leptin resistance. In the gut, GF mice demonstrated heightened expression of anorexigenic gut hormones, including peptide YY (Pyy) and cholecystokinin (Cck), in addition to increased levels of G protein‐coupled receptors (GPCRs) involved in gut hormone secretion and nutrient metabolism, particularly in females. While carbohydrate metabolism genes were upregulated in CON mice, lipid metabolism genes were predominantly higher in GF mice. These findings suggest that the gut microbiota downregulates genes involved in appetite suppression, modulates GPCRs linked to gut hormone secretion, and contributes to leptin resistance, particularly in males. This research underscores the importance of the gut microbiome in host metabolism and reveals potential molecular targets for novel treatments of metabolic diseases.

## 1 INTRODUCTION

The human body holds trillions of microorganisms, comprising fungi, viruses, bacteria, and protozoa, known as the gut microbiome (GMB). Most of these organisms live symbiotically with their host and play a role in breaking down complex fibers found in the diet, and by‐products of this microbial metabolism include metabolites such as butyrate (Valdes et al., 2018). The GMB and its metabolites are considered to be essential components of many physiological processes, such as metabolism, immunity, and behavior (Valdes et al., 2018). Importantly, the composition of the GMB is highly dynamic and can be significantly influenced by external factors, such as host diet and lifestyle (Spor et al., 2011). Changes in GMB have been implicated in the pathophysiology of various human metabolic and neurological diseases through the brain‐gut axis (Agirman & Hsiao, 2021; Keogh, Kim, et al., 2021; Keogh, Rude, & Gareau, 2021; van Son et al., 2021). Further, human and animal studies indicate the existence of an effect of sex on GMB composition as well as metabolism and brain function (Holingue et al., 2020; Valeri & Endres, 2021).

Due to the vital influence of the GMB on various aspects of metabolism, it is important to understand its contribution to the pathophysiology of the metabolic diseases, such as diabetes mellitus (DM) and obesity. These costly chronic diseases have high prevalence. According to the International Diabetes Federation Diabetes Atlas, as of 2021, 1 in 10 adults are living with (DM) worldwide, and this is predicted to increase further. A common comorbidity of type 2 diabetes mellitus (T2DM) is obesity, underscoring the importance of understanding the underlying mechanisms of these interconnected metabolic diseases. Obesity is characterized by increased adiposity, leptin resistance, chronic low‐grade inflammation, and aberrant energy homeostasis (van Son et al., 2021; Yao et al., 2020). Perturbations in gut microbiome composition, such as increased *Firmicutes* and decreased *Bacteroidetes*, as well as increased gut permeability, reduced microbial diversity, and increased energy storage, are common in individuals with obesity (Muscogiuri et al., 2019; Schele et al., 2013; van Son et al., 2021). Importantly, previous studies have reported sexual dimorphism in the development of obesity, T2DM, and gut microbiome composition.

Previous studies investigated the effects of the microbiome on fat‐regulating neuropeptides and circulating leptin by evaluating gene expression in the brains of male conventional (CON) and germ‐free (GF) mice (Schele et al., 2013). The gut microbiome was associated with a relative increase in leptin resistance in male CON mice, leading to higher body weight and adiposity seen in CON mice when compared to GF mice (Schele et al., 2013). However, the effects of the microbiome on gut peptides, nutrient metabolism, and inflammation were not elucidated. Further, it is unclear whether these factors exhibit a sexual dimorphic nature. In the present study, we aimed to characterize neuroendocrine and enteroendocrine genes important for metabolic health that may be impacted by the presence or absence of the gut microbiome. We used the gene expression of certain G‐protein coupled receptors (GPCRs) important for metabolism to help elucidate the impact of the GMB on nutrient digestion and gut hormone secretion. We hypothesized that the presence of the microbiome could regulate metabolism through gene expression changes in the neuroendocrine and enteroendocrine systems in a sex‐dependent context. Understanding the role of the gut microbiome in the function of these genes may be critical for the development of microbiome‐relevant novel therapeutics for metabolic disease.

## 2 MATERIALS AND METHODS

### 2.1 Experimental animals

Thirty‐six 14‐week‐old C57BL/6 male and female mice were used in this study; 18 were germ‐free (GF), born and raised in a sterile environment, and eighteen were conventionally raised (CON) with a naturally occurring microbiome (*n* = 9/sex/condition). The germ‐free mouse colony, maintained at Purdue University, was originally purchased from Charles River Laboratories (Wilmington, MA), and the conventionally raised mice used in the current study were also obtained from the same vendor. After arrival, the age‐matched conventional cohort of mice was allowed to acclimate for a week in the Lab Animal Resource Center (LARC) at Indiana University School of Medicine. The mice were housed in a 12‐hour light/12‐h dark cycle with light on at 7 am and off at 7 pm each day. Mice of the same sex were group housed with 4 to 5 mice housed in one cage. Female mice were not synchronized in their estrous cycles. Both cohorts were ad libitum fed a standard chow diet (autoclaved Teklad Global 18% Protein Rodent Diet, Envigo 2018S). Mice were euthanized via CO_2_ asphyxiation.

### 2.2 Animal studies and ethic approval

The study was exempt from Institutional Review Board (IRB) approval because no human subjects were involved in the study. The ethic approval for animal studies was approved as stated here. All animal procedures were followed according to the Indiana University School of Medicine Institutional Animal Care and Use Committee (IACUC #11121, 19,013, 22,006) and Purdue University Institutional Animal Care and Use Committee (IACUC #1909001951).

### 2.3 Tissue collection

The mediobasal hypothalamus (MBH), duodenum, jejunum, ileum, and colon were collected immediately following euthanasia. The gut and brain dissections were performed as previously described (Yan et al., 2022). Briefly, 1 cm segments were dissected from the duodenum (adjacent to the pyloric sphincter), jejunum (halfway between the pyloric sphincter and the ileocecal valve), ileum (3 cm above the ileocecal valve), and colon (halfway between the anus and the cecocolic junction). All tissues were stored at −80°C until processing (Yan et al., 2022).

### 2.4 RNA quantification

RNA samples were extracted from the tissues using Trizol reagent (Life Technologies, catalog #15596018, Carlsbad, CA, USA). The gut sections were reconstituted in 300 μL of nuclease‐free water, and the MBH was reconstituted in 18 μL of nuclease‐free water. The tissues underwent reverse transcription polymerase chain reaction (RT‐PCR), with a total RNA input of approximately 1136 ng for the MBH, 5000 ng for the duodenum and jejunum, 1000 ng for the ileum, and 1600 ng for the colon (Invitrogen, SuperScript First Strand, catalog #11904018, Carlsbad, CA, USA). The transcription levels of *ActB*, *Agrp*, *Pomc*, *Npy*, *Gpr17*, *Socs3*, *Gpr120*, *Gpr119*, *Cck*, *Pyy*, *Gip*, *Gcg*, *ApoB*, *Ffar3*, *Pck2*, *Sis*, *Fatp4*, *Glut2* were measured by quantitative PCR (qPCR) with the GoTaq qPCR Master kit (Promega, catalog #A6002, Madison, WI, USA), as previously described (Reilly et al., 2022). The primers were standardized to the expression levels of β‐actin for each tissue, and these values were normalized to the male duodenum expression levels to compare the various groups. The primer sequences are included in Table S1.

### 2.5 Serum biochemistries

Mice were ad libitum fed and subjected to blood sampling in the morning. Blood samples were collected via cardiac puncture. Serum insulin was measured by enzyme‐linked immunosorbent assay (ELISA) (Mercodia AB, catalog #10–1247‐10, Uppsala, Sweden). Serum leptin was also measured by ELISA (EMD Millipore, catalog #EZML‐82 K, Burlington, MA, USA). All reactions were performed according to manufacturer protocols.

### 2.6 Statistical analysis

Data was analyzed with an unpaired *t*‐test or two‐way ANOVA and Sidak's Multiple Comparisons Test using GraphPad Prism software (Boston, MA, USA), as specified in figure legends. The factors considered in the two‐way ANOVA analysis were microbiome status (GF vs. CON), sex, and gut segment (duodenum, jejunum, ileum, colon). All analyses used *p* < 0.05 as a threshold to determine significance.

## 3 RESULTS

### 3.1 Germ‐free mice have increased expression of *Pomc* and *Npy*, and decreased expression of *Socs3* in the hypothalamus

**FIGURE 1:**
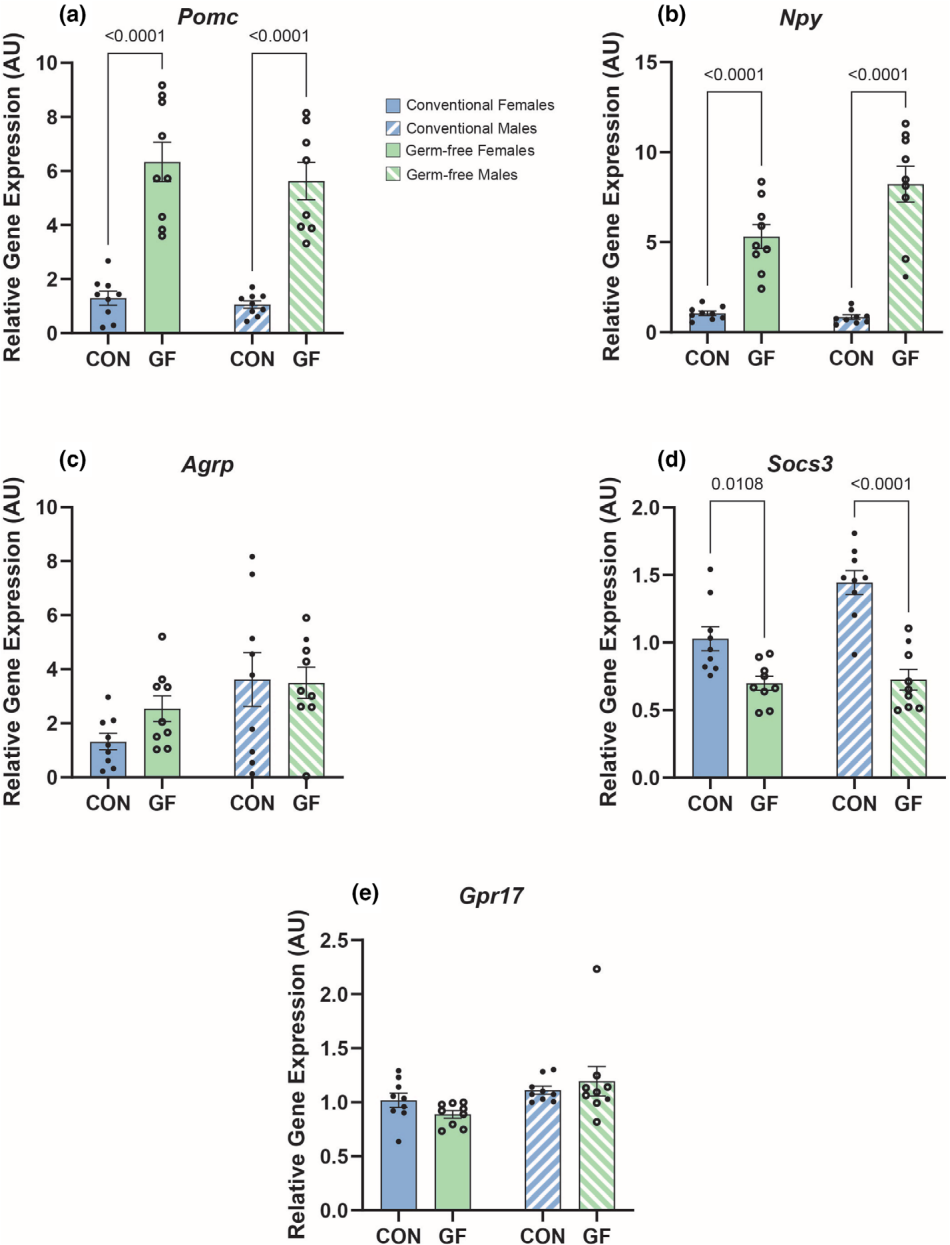
Variations in metabolic gene expression in the medial basal hypothalamus of germ‐free and conventional mice. Relative gene expression of *Pomc* (a), *Npy* (b), *Agrp* (c), *Socs3* (d), and *Gpr17* (e) in the medial basal hypothalamus (MBH) of germ‐free and conventional mice. Data represent mean ± SEM and were analyzed by two‐way ANOVA and Sidak's Multiple Comparisons Test with *p* values labeled in the graph.

Our results showed that, in the medial basal hypothalamus (MBH), GF mice had significantly increased expression of neuropeptides associated with feeding regulation, that is, *Proopiomelanocortin* (*Pomc*) and *Neuropeptide Y* (*Npy*), compared to CON mice (Figure 1a,b). Conversely, there was no difference between the levels of *Agouti‐related peptide* (*AGRP*) expression for CON and GF mice (Figure 1c). Furthermore, CON mice had significantly higher expression of a negative regulator of leptin signaling, *Suppressor of cytokine signaling 3* (*Socs3*) in the MBH (Figure 1d). Additionally, we detected a greater expression of *Agrp* and *Socs3* in the CON males compared to the CON females (Figure S1c,d). Though *G protein‐coupled receptor 17* (*Gpr17*) expression was comparable between CON and GF mice (Figure 1e), it displayed sexual dimorphism in the GF mice, with males having increased expression compared to females (Figure S1e).

### 3.2 Germ‐free mice display aberrant expression levels for genes encoding gut hormones and the receptors that regulate gut hormone secretion

**FIGURE 2:**
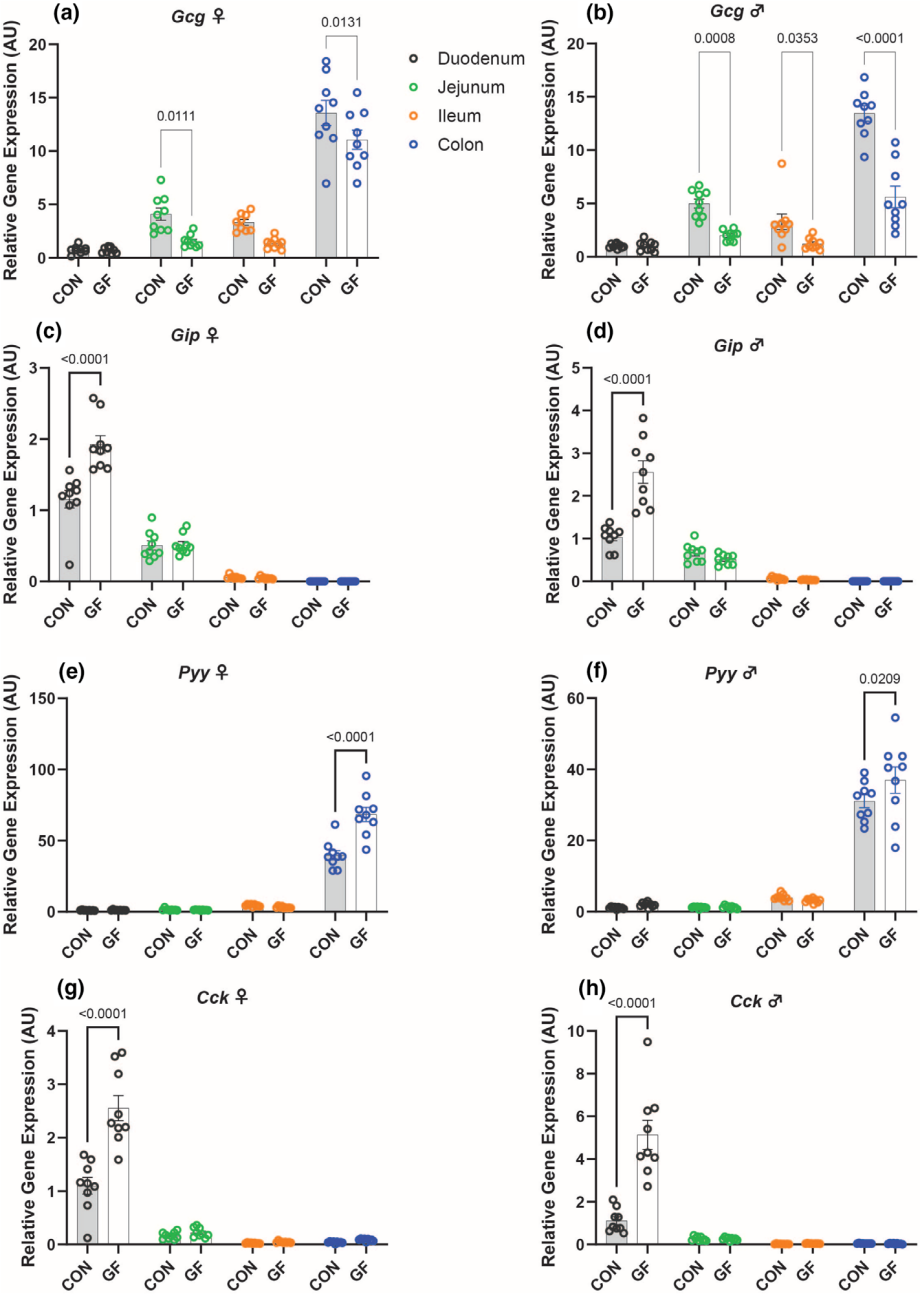
Variations in Gut Hormone Expression Levels in Conventional and Germ‐Free. Relative gene expression of *Gcg* (a, b), *Gip* (c, d), *Pyy* (e, f), and *Cck* (g, h) in the gut (duodenum, jejunum, ileum, colon) of germ‐free and conventional mice. Data represent mean ± SEM and were analyzed by two‐way ANOVA and Sidak's Multiple Comparisons Test with *p* values labeled in the graph.

**FIGURE 3:**
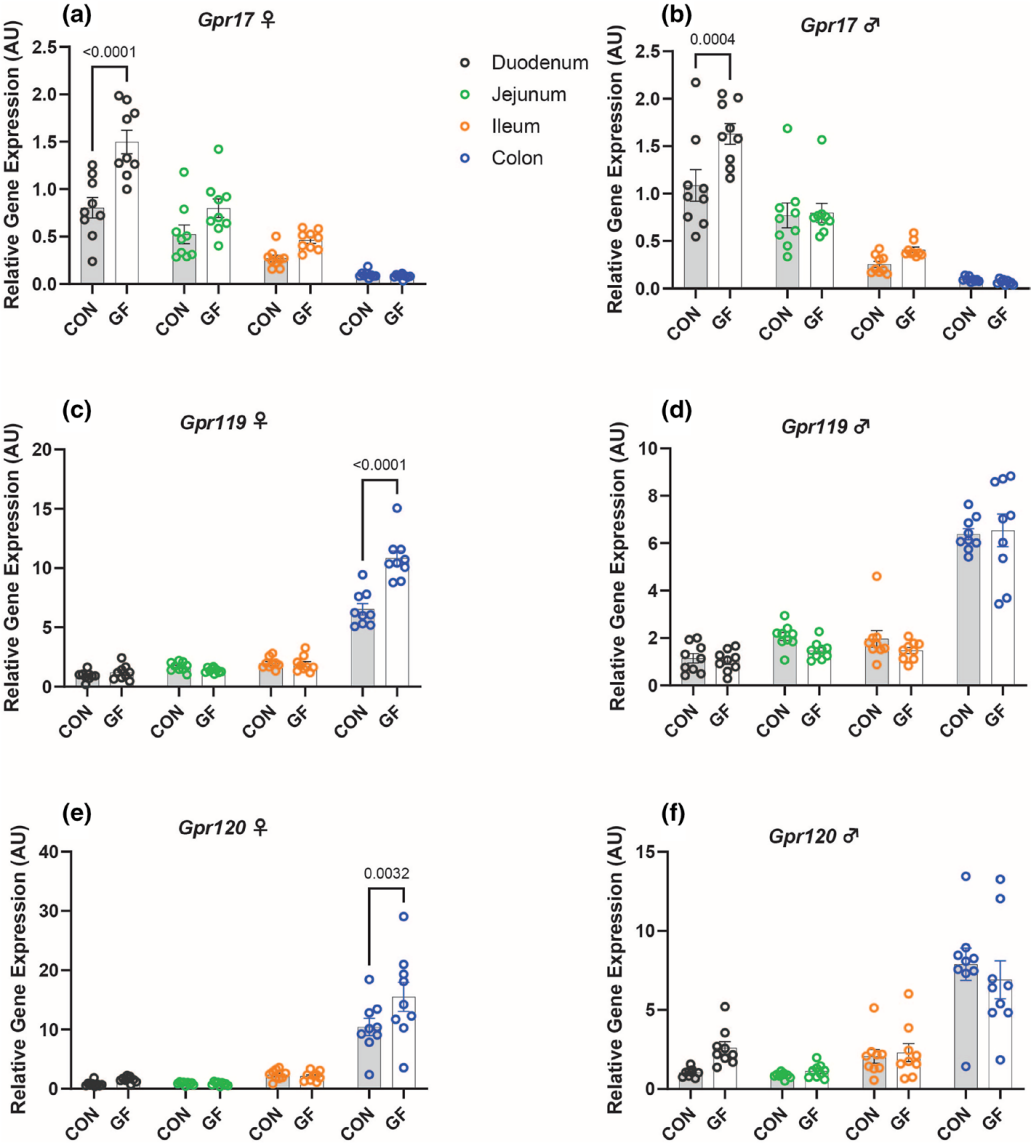
Variations in GPCR Gene Expression Levels in Conventional and Germ‐Free. Relative gene expression *Gpr17* (a, b), *Gpr119* (c, d), and *Gpr120* (e, f) in the gut (duodenum, jejunum, ileum, colon) of germ‐free and conventional mice. Data represent mean ± SEM and were analyzed by two‐way ANOVA and Sidak's Multiple Comparisons Test with *p* values labeled in the graph.

Interestingly, the absence of a microbiome had differing effects on the expression of incretin hormones, which are secreted after meal ingestion, and the GPCRs that regulate their secretion. For example, in the jejunum and colon of CON mice, the expression of g*lucagon* (*Gcg*), a precursor to *glucagon‐like peptide 1* (*Glp‐1*), was significantly increased for both males and females compared to that of GF mice. In contrast, *glucose‐dependent insulinotropic polypeptide* (*Gip*) showed increased expression in the duodenum of male and female GF mice (Figure 2a–d). Further, GF mice demonstrated increased expression of genes related to the anorexigenic gut hormones, *peptide yy* (*Pyy*) and *cholecystokinin* (*Cck*) in the duodenum and colon, respectively, for both male and female mice (Figure 2e–h). We examined the expression of GPCRs that either stimulate or inhibit gut peptide secretion. *G protein‐coupled receptor 119* (*Gpr119*) and *G protein‐coupled receptor 120* (*Gpr120*) showed increased expression in the colon of female GF mice compared to that of CON female mice (Figure 3c–f). *Gpr17* was found to be higher in the duodenum of the GF cohort (Figure 3a,b).

In the colon, GF females display higher expression levels of *Gcg*, *Pyy*, *Gpr119*, and *Gpr120* compared to GF males (Figures S2b,f and S3d,f). Similarly, in the colon of the CON mice, females demonstrated increased expression of *Pyy* and *Gpr120* compared to males (Figures S2e and S3e). For the GF mice, males show increased expression of *Gip* and *Cck* (Figure S2d,h). This pattern is not reciprocated in the CON mice.

**FIGURE 4:**
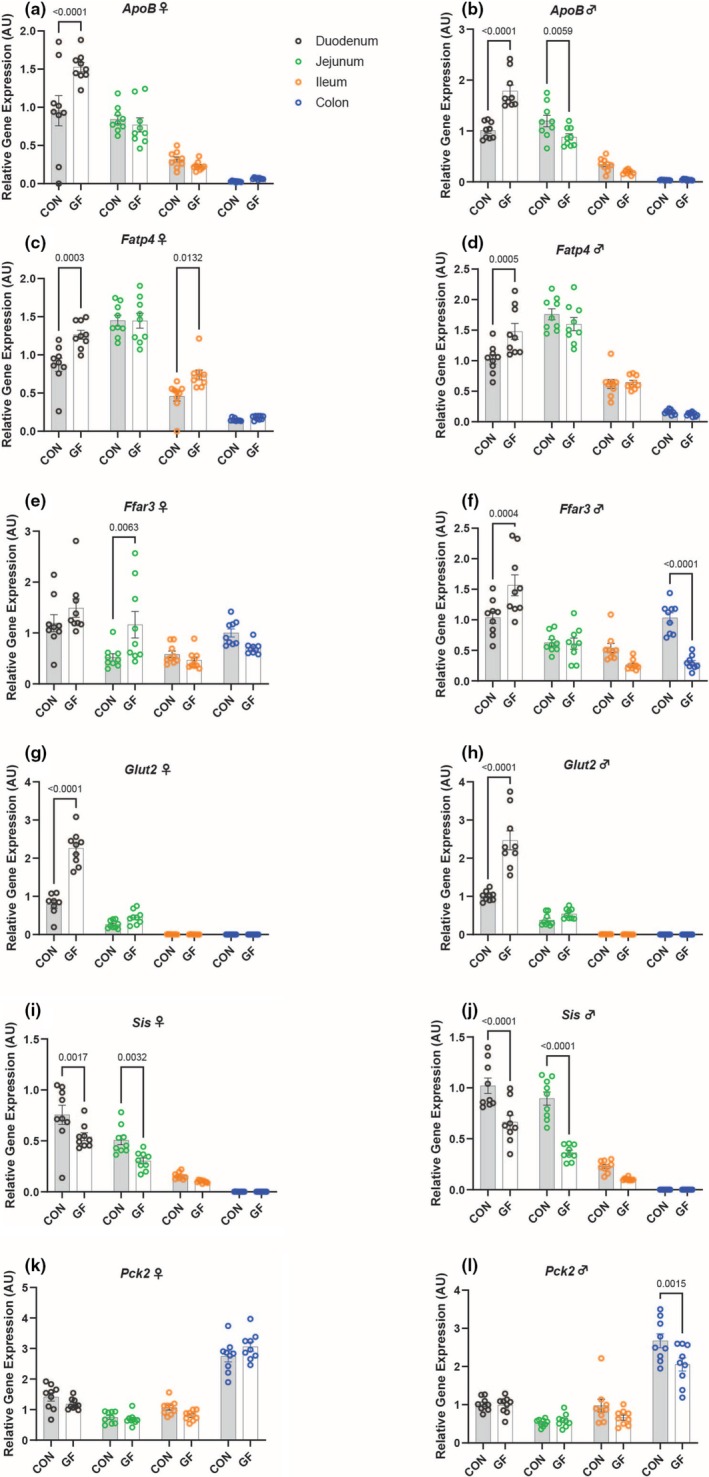
Variations in Nutrient Metabolism Expression Levels in Conventional and Germ‐Free. Relative gene expression of *ApoB* (a, b), *Fatp4* (c, d), *Ffar3* (e, f), *Glut2* (g, h), *Sis* (i, j), and *Pck2* (k, l) in the gut (duodenum, jejunum, ileum, colon) of germ‐free and conventional mice. Data represent mean ± SEM and were analyzed by two‐way ANOVA and Sidak's Multiple Comparisons Test with P values labeled in the graph.

To investigate carbohydrate and fatty acid metabolism, we looked at relative expression levels of *Apolipoprotein B* (*ApoB*), *Fatty acid transporter 4* (*Fatp4*), *Free fatty acid receptor 3* (*Ffar3*), *Glucose transporter 2* (*Glut2*), *Sucrase isomaltase* (*Sis*), and *Phosphoenolpyruvate carboxykinase 2* (*Pck2*). Germ‐free females had increased expression in the duodenum for *ApoB*, *Fatp4*, and *Glut2* compared to CON females (Figure 4a,c,g). Germ‐free females also had increased expression of *Fatp4* in the ileum and *Ffar3* in the jejunum (Figure 4c,e). Conversely, CON females had increased expression of *Sis* in the duodenum and jejunum compared to GF females (Figure 4i). In male mice, GF mice had increased expression in the duodenum for *ApoB*, *Fatp4*, *Ffar3*, and *Glut2* (Figure 4b,d,f,h). In the colon, GF males also demonstrated increased expression of *Ffar3* (Figure 4f). The CON males had increased expression of *ApoB* in the jejunum, *Sis* in the duodenum and jejunum, and *Pck2* in the colon compared to GF males (Figure 4b,k,l).

For *ApoB*, *Fatp4*, and *Sis*, CON males display higher gene expression levels in the jejunum than CON females (Figure S4a,c,i). However, the only significant difference between male and female GF mice in the jejunum is seen in *Ffar3*, where the females have greater gene expression than the males (Figure S4f). In the duodenum, CON males show increased expression of *Glut2* and *Sis* compared to CON females (Figure S4g,i). For GF mice, the males demonstrated greater expression of *ApoB* and *Sis* in the duodenum (Figure S4b,j). Additionally, in the colon of GF mice, females demonstrated increased expression of *Pck2* (Figure S4l).

### 3.3 Male germ‐free mice have decreased serum leptin compared to conventional male mice

**FIGURE 5:**
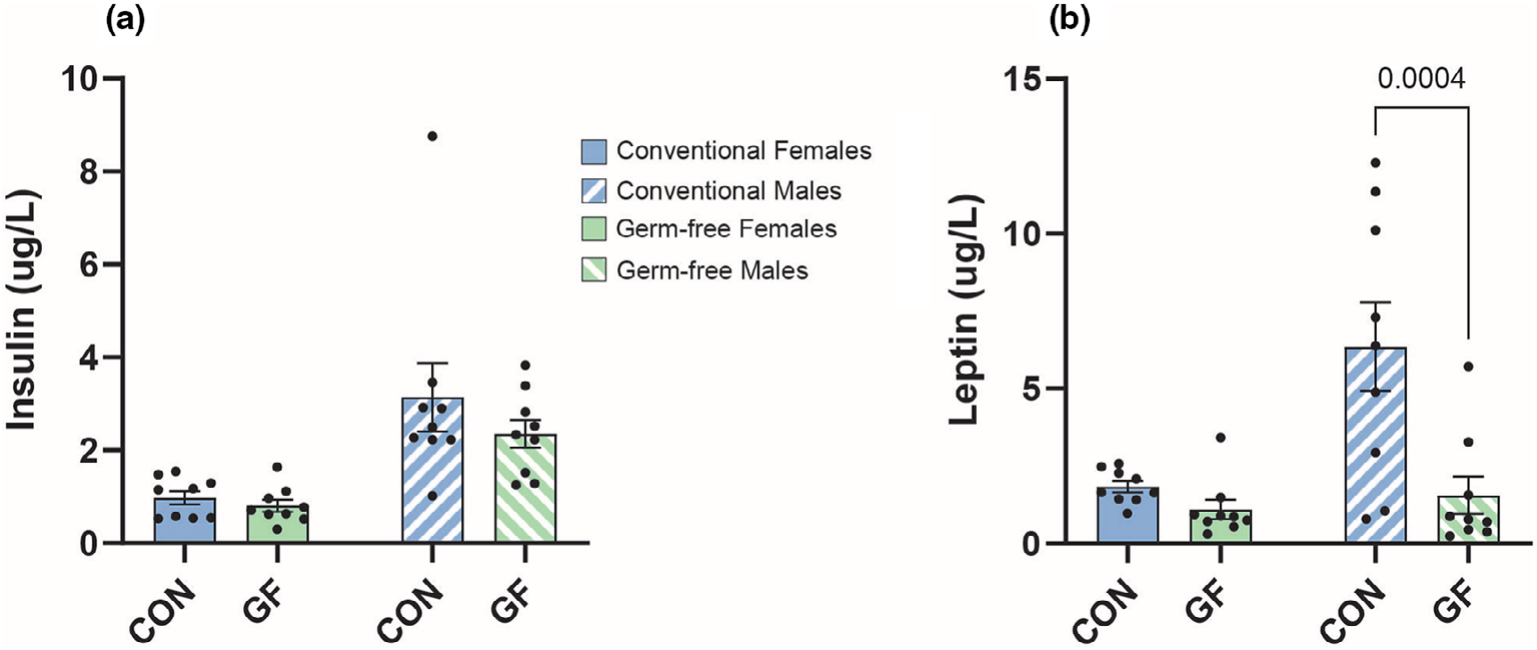
Variations in Insulin and Leptin Levels for Germ‐free and Conventional Mice. Concentrations of serum insulin (a) and leptin (b) for conventional and germ‐free mice. Data represent mean ± SEM and were analyzed by two‐way ANOVA with Sidak's Multiple Comparisons Test with *p* values labeled in the graph.

There was no significant difference in circulating insulin between the CON and GF mice, although the insulin concentrations of the CON mice trended higher than that of GF mice, regardless of sex (Figure 5a). Interestingly, in both GF and CON, males had significantly higher levels of serum insulin than the females (Figure S5a,c). Leptin was measured via ELISA in the serum of GF and conventional mice. GF males showed significantly increased levels of leptin compared to CON males (Figure 5b). However, no differences were noted between female GF and CON mice. Interestingly, a sexually dimorphic response was observed between the CON male and female mice. The males had approximately three times more circulating leptin than the females (Figure S5b). Germ‐free mice did not display any statistically significant sexual dimorphic effects, although the males trended higher than the females (Figure S5d). This suggests that the gut microbiome may be a critical factor in the sexual dimorphism of circulating leptin.

## 4 DISCUSSION

We aimed to characterize neuroendocrine and enteroendocrine genes important for metabolic health that may be impacted by the presence or absence of the gut microbiome. Presently, we report that, in the hypothalamus, GF mice displayed increased expression of the anorexigenic gene *Pomc* as well as a decrease in the expression of *Socs3*, a negative regulator of leptin signaling. Furthermore, the CON males had significantly more serum leptin than the GF males. In the gut, we observed variations in gene expression for gut hormones, GPCRs, and nutrient metabolism and inflammatory markers between GF and CON mice, regardless of sex. These differences were almost exclusively in the duodenum and colon, with few differences observed in the jejunum and ileum. For the genes involved in carbohydrate metabolism, a majority were higher in the CON compared to GF, whereas for lipid metabolism, we observed an opposite effect. For the genes involved in gut hormone secretion, there was an increase in *Gcg* expression, a precursor to the incretin hormone *glucagon‐like peptide 1* (*Glp‐1*), in the CON mice compared to the GF mice. Conversely, there was a decrease in the expression levels of *Gip*, an incretin hormone implicated in lipid metabolism, in both CON males and females. Two additional anorexigenic gut hormones, *Pyy* and *Cck*, were also decreased in the CON mice. Female GF mice had increases in all 3 GPCRs tested, with *Gpr17* in the duodenum and *Gpr119* and *Gpr120* in the colon. Lastly, the GF mice had increases in multiple inflammatory markers in both males and females.

Both the neuroendocrine and enteroendocrine systems play important roles in metabolism, energy homeostasis, and nutritional status. These two systems work in concert to regulate metabolism in a connection termed the gut‐brain axis. Our evidence indicated that the gut microbiome upregulated genes involved in carbohydrate metabolism and leptin homeostasis and downregulates genes involved in lipid metabolism and anorexigenic signaling. Furthermore, our results suggest that the gut microbiome contributes to leptin resistance through *Socs3* upregulation. SOCS3 is a crucial inhibitor of leptin signaling. It is transcriptionally upregulated by leptin signaling and functions by preventing JAK2 activation in the leptin signaling pathway (Bjorbaek et al., 1998). Previously, the gut microbiome has been indicated to interplay with *Socs3* gene expression and function in conditions such as myeloid hematopoiesis and obesity (Cho et al., 2021; Deng et al., 2019).

The evidence in the present study also corroborates findings previously reported that leptin resistance and neuropeptides involved in feeding regulation are partially controlled by gut microbiome (Schele et al., 2013). The gut microbiome has been previously associated with a relative increase in leptin resistance, body weight, and adiposity in CON mice when compared to GF mice (Schele et al., 2013). In the 2013 study, they found decreases in expression levels of *Npy* and *Agrp*, as well as increased expression levels of *Pomc* in the hypothalamus of male CON mice compared to GF mice. Similarly, we found that both male and female CON mice display decreased hypothalamic levels of *Npy*, but we also saw a decrease in the levels of *Pomc*, with no significant differences in *Agrp* expression. The increase in both anorexigenic and orexigenic neuropeptides of GF mice is likely compensatory, in order to maintain energy homeostasis in the absence of a microbiome. Thus, it suggests that these neuropeptides have a dynamic interplay with gut microbiome. Importantly, we observed an increase in the hypothalamic expression of *Socs3*, a gene that encodes a downstream inhibitor of leptin signaling, in both male and female CON mice, as well as significantly higher serum leptin in the CON males. *Socs3* has previously been reported to be a critical mediator of leptin resistance. Thus, we believe that this increase in *Socs3* may be a major contributor to GMB‐mediated leptin resistance.

Gut hormones are secreted from enteroendocrine cells (EECs) in the intestinal epithelium upon stimulation by food ingestion. As a result of this function, gastrointestinal (GI) hormones play a vital role in regulating energy, appetite, and glucose homeostasis (Xie et al., 2020). The incretin hormones, *Glp‐1* and *Gip*, contribute to the increase in postprandial glucose‐stimulated insulin secretion from the pancreatic β‐cells, a process called the incretin effect. This process improves glucose tolerance, allowing for the ingestion of larger amounts of glucose without a subsequent increase in glucose excursion (Holst et al., 2021). The incretin effect also promotes satiety and gastric emptying. Presently, we report an increase in the expression levels of *Gcg* in the jejunum and colon of female CON mice and the jejunum, ileum, and colon of male CON mice compared to the sex‐matched GF mice. However, there was an increase in the relative gene expression of *Gip* in the duodenum of male and female GF mice compared to the male and female CON mice. Although both *Glp‐1* and *Gip* play important roles in energy and glucose metabolism, the exact function of each hormone individually has been contested. The anorexigenic effects of *Glp‐1* are more widely supported, while the effect of *Gip* on food intake, although currently unclear, has been suggested to be minimal (Holst & Rosenkilde, 2020). We also did not observe a difference in the serum levels of insulin between GF and CON mice, although the males showed significantly higher insulin levels compared to the females in their respective microbiome status.

Furthermore, the two anorexigenic gut hormones, *Cck* and *Pyy*, were increased in the duodenum and colon, respectively, of the GF mice compared to the CON mice. Both *Cck* and *Pyy* are primarily stimulated by the consumption of proteins and lipids, and they aid in the regulation of digestion and nutrient absorption by inducing satiety (Miller et al., 2021). Interestingly, when looking at *Gpr119* and *Gpr120*, two GPCRs that are stimulated by dietary free fatty acids (FFAs) and stimulate the secretion of incretin hormones, we see that they are significantly upregulated in the colon of GF females compared to both GF males and CON females.

Gut microbes and their metabolites, such as short chain fatty acids, can modulate gut hormone secretion (Gurung et al., 2020; Muscogiuri et al., 2019). In particular, it has been shown that *Free fatty acid receptor 3* (*Ffar3—*also known as *Gpr41*) interacts with short chain fatty acids produced by the gut microbiota (Muscogiuri et al., 2019). It is speculated that the activation of this receptor stimulates the release of *Pyy*, an anorexigenic hormone, and leptin, reducing food intake (Muscogiuri et al., 2019; Samuel et al., 2008). GF mice displayed increases in expression for *Pyy* in the colon of males and females, as well as increases in *Ffar3* in the jejunum of the GF females and duodenum of the GF males. Conversely, we do see higher expression of *Ffar3* in the colon of male GF mice compared to that of CON mice. Given the results of the 2008 study by Samuels and colleagues (Samuel et al., 2008), it makes sense that we would see an increase of *Ffar3* in the colon of CON males to counteract the decrease in *Pyy* in the male colon.

GPCRs are key players in the brain‐gut axis by acting as sensors for neurotransmitters, peptide hormones, nutrients, and metabolites present in the gut lumen and systemic circulation. Human GPCRs can sense microbial metabolites to modulate host physiology (Chen et al., 2019; Colosimo et al., 2019). The studies from our group, as well as other groups, show that an orphan GPCR, GPR17, regulates metabolic homeostasis in the brain hypothalamic neurons and oligodendrocytes (Conley et al., 2021; Ou et al., 2019; Reilly et al., 2019; Ren et al., 2012; Ren et al., 2015). Moreover, we showed inhibiting GPR17 action in the intestinal enteroendocrine cells increased incretin hormone GLP‐1 secretion (Conley et al., 2024; Yan et al., 2022; Zhu, 2024). The endogenous ligand for GPR17 remains elusive. It is likely GPR17 responds to chemical cues derived from microbial products. We found that *Gpr17* expression is upregulated in the duodenum of GF mice, which could reflect a compensatory increase in the absence of gut microbes, but further investigation is needed to determine which microbes and metabolites interact with *Gpr17*. Although the present study determined that there were differences in gene expression levels between GF and CON mice, future investigations into whether functional changes accompany these variations in gene expression are warranted. Additionally, it would be interesting to see if, after colonization with the stool of CON mice, the gene expression of GF mice changes to match that of the conventional mice. Further, future studies are warranted to investigate the effect of metabolic gene expression after the introduction of specific microbes known to produce certain metabolites or influence digestion of certain nutrients.

Previously, the gut microbiome has been proposed to be involved in the pathogenesis of obesity through increased carbohydrate fermentation and energy utilization, later being stored as excess fat or glucose (Muscogiuri et al., 2019). The gene *Pck2* encodes a mitochondrial enzyme integral to gluconeogenesis, the process of converting non‐carbon substrates into glucose (Yu et al., 2021). It has been found that an increase in the activity of *Pck2* leads to exacerbation of diabetes mellitus by increasing glucose production (Yu et al., 2021). Similarly, the gene *Sis* plays an important role in the digestion of certain starches and carbohydrates, such as sucrose and maltose, for absorption by the intestine (Cohen, 2016). Presently, we found that the male CON mice had increased gene expression for both *Pck2* (colon) and *Sis* (duodenum and jejunum) compared to GF males, and the female CON mice had an increase in *Sis* (duodenum and jejunum) compared to GF females. Conversely, GF mice had increased expression of *Glut2*, a transmembrane protein that facilitates the transfer of glucose across the cell membrane, in the duodenum of both males and females compared to the CON mice. It has been suggested that intestinal *Glut2* knock‐out mice display improvements in gut barrier function and inflammation (Schmitt et al., 2017). Thus, a decrease in *Glut2* expression in CON mice may be protective against dysbiosis by enhancing the gut barrier, a feature that would not be as integral to GF mice, as they do not have bacteria that would cross the gut barrier. The increase in *Glut2* in GF mice may also contribute to differences in gut inflammatory markers. For example, increased *Glut2* may lead to increased inflammation and thus higher expression of genes involved in immunity and inflammation.

## 5 LIMITATIONS OF THE STUDY

There are limitations with our current studies. First, the mechanisms mediating the gene expression changes in the neuroendocrine and enteroendocrine systems are still elusive. Which gut microbe or microbial metabolites contribute to the changes is unclear. Moreover, how the lack of or re‐colonization of the germ‐free animals with certain microbes could change body weight, adiposity, food intake, and glycemia is a set of interesting questions. Future mechanistic studies to address these questions are warranted. Second, although we observed the changes in gut hormones and GPCRs gene expression related to nutrient metabolism in a sexual dimorphic manner, the role of sex hormones is still unclear. It is very possible that the observed sexual dimorphism reflects an adaptive response to the absence of microbiota that differs between males and females or, alternatively, a microbiota‐regulated hormonal mechanism. Our group has data indicating differences in sex hormone levels between germ‐free and conventional mice, suggesting a potential role for the gut microbiome in regulating these hormones, which could further impact the expression of these genes presented in the current report. We measured kisspeptin (KISS1) gene expression from the arcuate nucleus in the hypothalamus (data not shown). This gene is the upstream regulator of hypothalamic signaling to initiate sex hormone production in the gonads. This data indicates that germ‐free mice exhibit inhibited signaling necessary for initiation of steroidogenesis and further suggests the gut microbiome is a critical regulator of sex differences in metabolic health. Though beyond the scope of this study, this presents an interesting avenue for future research. Third, although we selected genes with key roles in regulating energy balance and nutrient absorption in the brain and gut of the GF and CON mice cohorts, we have yet to decipher the mechanisms governing the gene expression changes and metabolic outcomes. A comprehensive transcriptional profiling of the neuroendocrine and enteroendocrine systems could generate a more information in addition to the targeted approach used in this study. Moreover, the current study focuses on the brain and gut, while the microbiome is known to mediate lipid metabolism. Other organs and pathways critical for lipid metabolism, such as the liver and fat, are warranted for future studies. Understanding the relationship between the microbiome and lipid metabolism in GF mice could be a crucial factor in the development of leptin resistance and body weight gain. Moreover, whether the observed expression changes in genes mediating nutrient absorption and gut hormone secretion are an adaptive change or a causal factor for the metabolic differences in GF and CON mice requires future studies.

## 6 CONCLUSION

The data presented in the current report supports that the gut microbiota may upregulate genes involved in the breakdown of carbohydrates, while downregulating those involved in the absorption of carbohydrates and lipid metabolism. Additionally, GF mice display increases in anorexigenic gut hormones and GPCRs compared to CON mice. Although CON mice do show an increase in *Gcg*, this increase may act as a compensatory measure to counteract the relative increase in carbohydrate metabolism found in CON mice. Gut microbes are known to assist in the host breakdown of otherwise insoluble carbohydrates, such as fiber, so it follows that the expression of certain genes involved in the breakdown of carbohydrates would decrease in GF mice, as they are not able to break these molecules down without the help of microbes. Furthermore, our results support the hypothesis that the gut microbiota modulates leptin resistance by upregulating the expression of *Socs3*. Our work offers insight for the development of novel therapeutics aimed at treating metabolic diseases, such as obesity and diabetes.

## AUTHOR CONTRIBUTIONS

Wynne Milhouse, Xun Sun, and Hongxia Ren designed and conducted experiments, analyzed data, and wrote and revised the manuscript. Anna Clapp Organski helped with tissue collection and manuscript revision. Hongxia Ren conceived and supervised the study. Tzu‐Wen L. Cross, Baohua Zhou, Xun Sun, and Derek Ai revised the manuscript. All authors reviewed and approved the manuscript.

## ETHICS STATEMENT

This study was conducted in accordance with ethical guidelines governing general laboratory practice including transparency, full disclosure, and integrity. The ethical use of animal models was approved and followed by the guidelines of Indiana University and Purdue University Animal Care and Use Committees.

## Supporting information


**Figure S1:** Sexual Dimorphism in Metabolic Gene Expression in the MBH. Relative gene expression of *Pomc* (a), *Npy* (b), *Agrp* (c), *Socs3* (d), and *Gpr17* (e) in the MBH of germ‐free and conventional mice. Two‐way ANOVA and Sidak’s Multiple Comparisons Test with *p < 0.05, **p < 0.01, ***p < 0.001, and ****p < 0.0001.

**Figure S2:** Sexual Dimorphism in Gut Hormone Gene Expression of Conventional and Germ‐free Mice. Relative gene expression of *Gcg* (a, b), *Gip* (c, d), *Pyy* (e, f), and *Cck* (g, h) in the gut (duodenum, jejunum, ileum, colon) of germ‐free and conventional mice. Two‐way ANOVA and Sidak’s Multiple Comparisons Test with *p < 0.05, **p < 0.01, ***p < 0.001, and ****p < 0.0001.

**Figure S3:** Sexual Dimorphism in GPCR Gene Expression of Conventional and Germ‐free Mice. Relative gene expression of *Gpr17* (a, b), *Gpr119* (c, d), and *Gpr120* (e, f) in the gut (duodenum, jejunum, ileum, colon) of germ‐free and conventional mice. Two‐way ANOVA and Sidak’s Multiple Comparisons Test with *p < 0.05, **p < 0.01, ***p < 0.001, and ****p < 0.0001.

**Figure S4:** Sexual Dimorphism in Nutrient Metabolism Expression Levels in Conventional and Germ‐Free. Relative gene expression of *ApoB* (a, b), *Fatp4* (c, d), *Ffar3* (e, f), *Glut2* (g, h), *Sis* (i, j), and *Pck2* (k, l) in the gut (duodenum, jejunum, ileum, colon) of conventional (a, c, e, g, I, k) and germ‐free (b, d, f, h, j, l) mice. Two‐way ANOVA and Sidak’s Multiple Comparisons Test with *p < 0.05, **p < 0.01, ***p < 0.001, and ****p < 0.0001.

**Figure S5:** Sexual Dimorphism in Insulin and Leptin Levels. Conventional male and female comparisons of insulin (a) and leptin (b). Germ‐free male and female comparisons of insulin (c) and leptin (d). Two‐way ANOVA with Sidak’s Multiple Comparisons Test (a, b) and unpaired t‐test (c‐f) with *p < 0.05, **p < 0.01, ***p < 0.001, and ****p < 0.0001. ROUT’s Test to Identify Outliers (a).

**Table S1:** Primer sequences used for qPCR. Each primer is listed with the gene, primer name, and DNA sequence. Each primer set defines which primer is the forward or reverse primer.

## Data Availability

The original contributions presented in this study are included in the article and the Supporting Information; further inquiries can be directed to the corresponding author. No AIGC was used for manuscript preparation.
