# Supplementary figures and images for: Microbiome affects mice metabolic homeostasis via differential regulation of gene expression in the brain and gut
Source: Physiol Rep. 2025 May 19;13(10):e70373. doi: 10.14814/phy2.70373 (PMC12087290; doi:10.14814/phy2.70373)

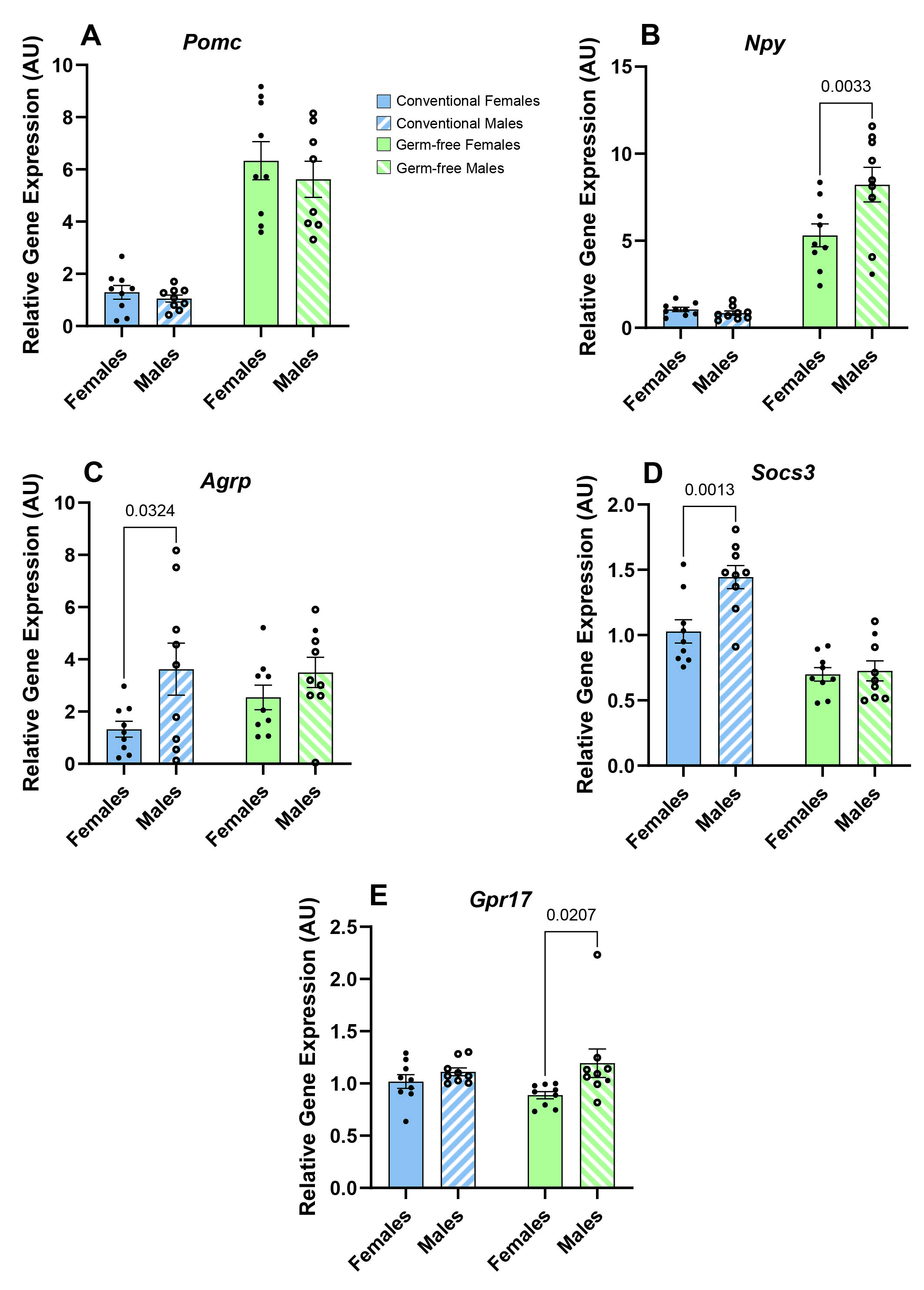

Supplement: Supplementary file 1 — Figure S1: Sexual Dimorphism in Metabolic Gene Expression in the MBH. Relative gene expression of Pomc (a), Npy (b), Agrp (c), Socs3 (d), and Gpr17 (e) in the MBH of germ‐free and conventional mice. Two‐way ANOVA and Sidak’s Multiple Comparisons Test with *p < 0.05, **p < 0.01, ***p < 0.001, and ****p < 0.0001. [file PHY2-13-e70373-s004.jpg]

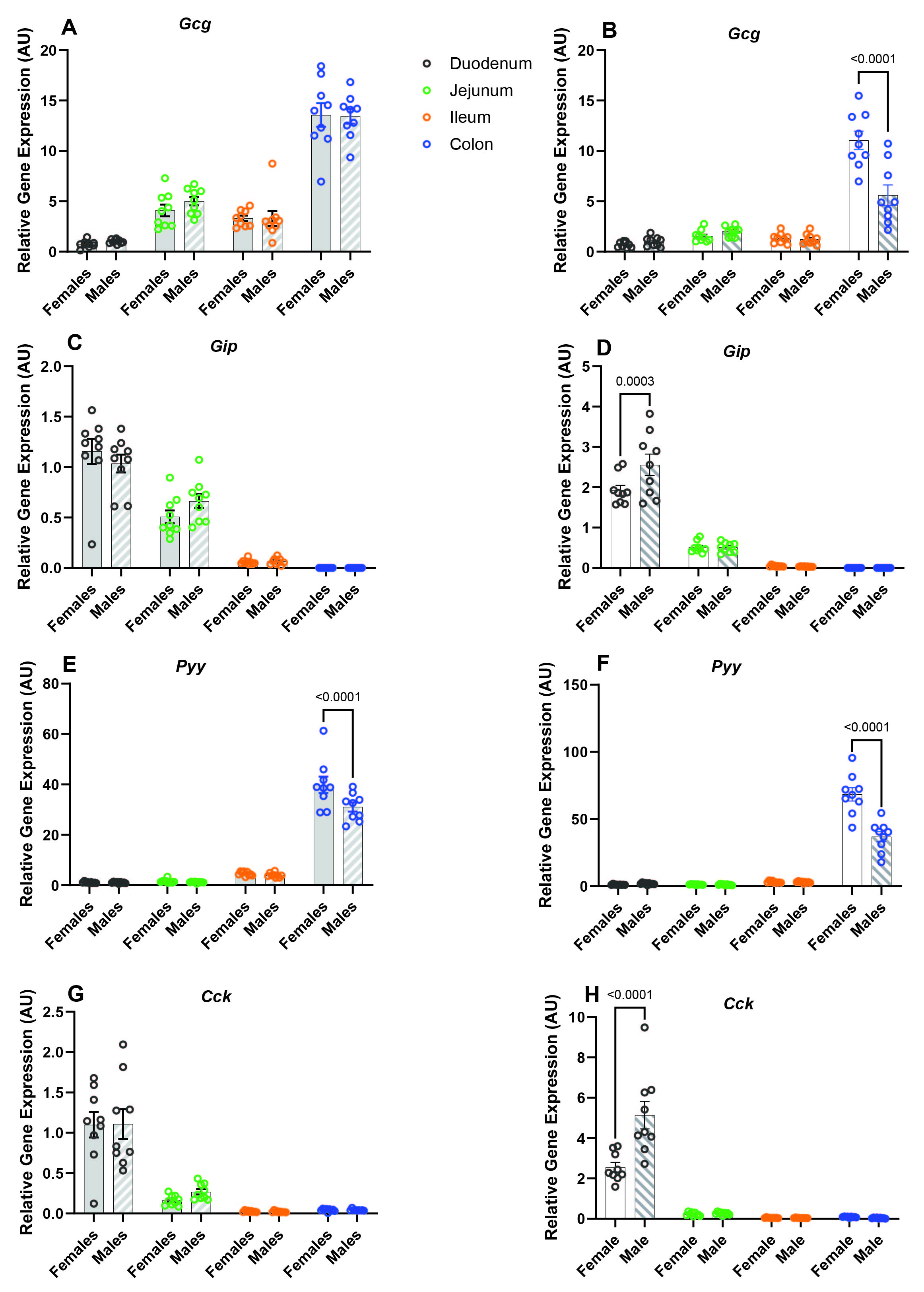

Supplement: Supplementary file 2 — Figure S2: Sexual Dimorphism in Gut Hormone Gene Expression of Conventional and Germ‐free Mice. Relative gene expression of Gcg (a, b), Gip (c, d), Pyy (e, f), and Cck (g, h) in the gut (duodenum, jejunum, ileum, colon) of germ‐free and conventional mice. Two‐way ANOVA and Sidak’s Multiple Comparisons Test with *p < 0.05, **p < 0.01, ***p < 0.001, and ****p < 0.0001. [file PHY2-13-e70373-s003.jpg]

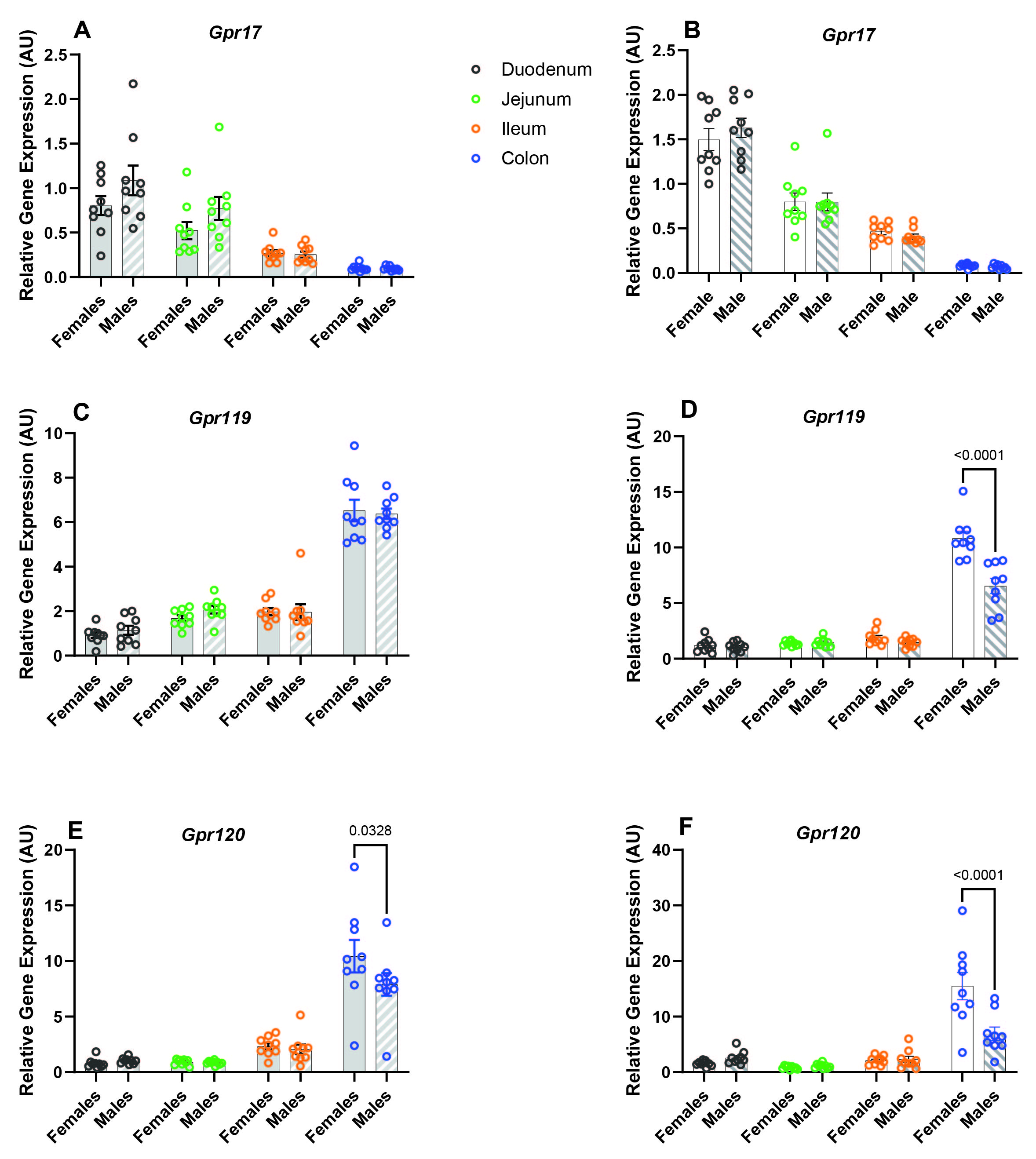

Supplement: Supplementary file 3 — Figure S3: Sexual Dimorphism in GPCR Gene Expression of Conventional and Germ‐free Mice. Relative gene expression of Gpr17 (a, b), Gpr119 (c, d), and Gpr120 (e, f) in the gut (duodenum, jejunum, ileum, colon) of germ‐free and conventional mice. Two‐way ANOVA and Sidak’s Multiple Comparisons Test with *p < 0.05, **p < 0.01, ***p < 0.001, and ****p < 0.0001. [file PHY2-13-e70373-s001.jpg]

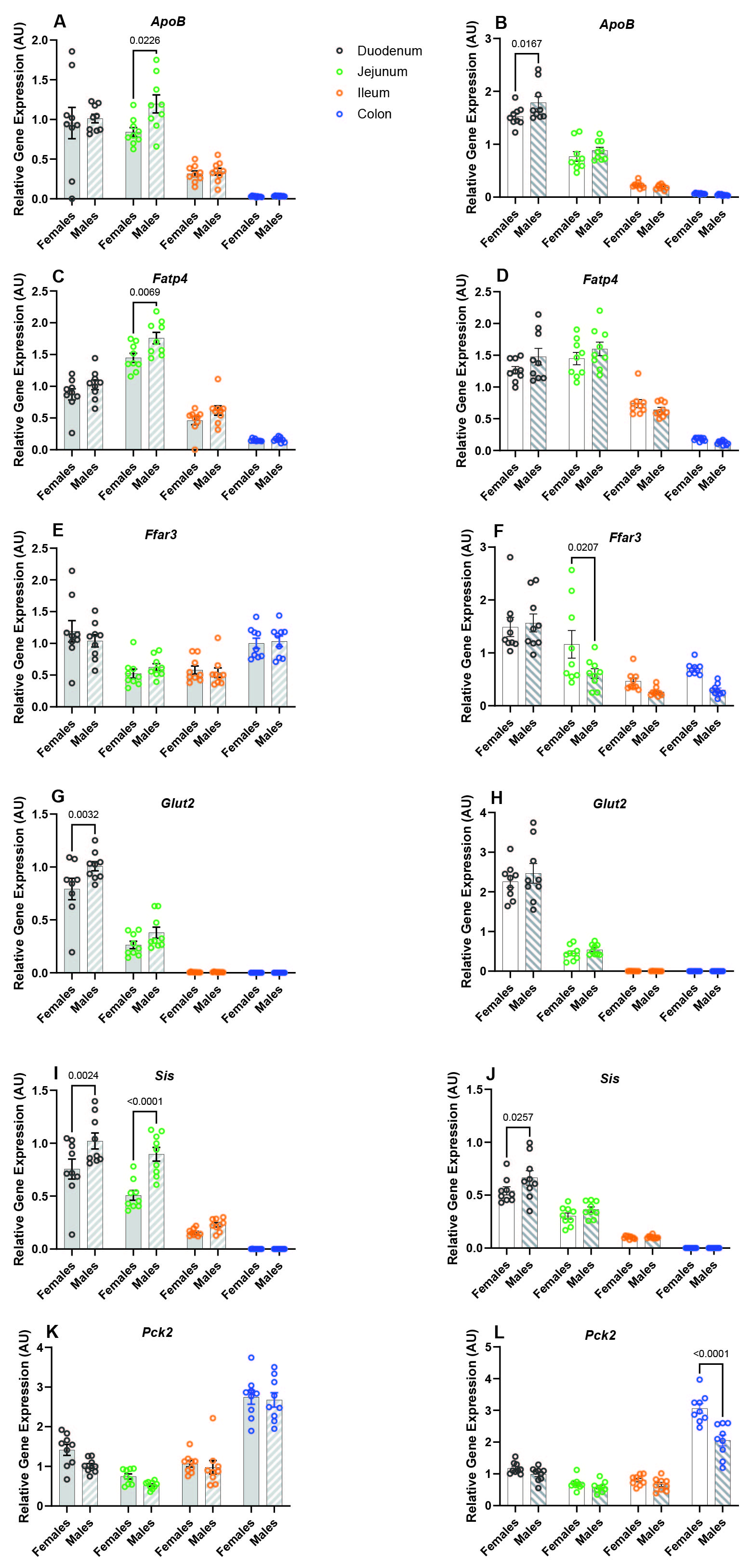

Supplement: Supplementary file 4 — Figure S4: Sexual Dimorphism in Nutrient Metabolism Expression Levels in Conventional and Germ‐Free. Relative gene expression of ApoB (a, b), Fatp4 (c, d), Ffar3 (e, f), Glut2 (g, h), Sis (i, j), and Pck2 (k, l) in the gut (duodenum, jejunum, ileum, colon) of conventional (a, c, e, g, I, k) and germ‐free (b, d, f, h, j, l) mice. Two‐way ANOVA and Sidak’s Multiple Comparisons Test with *p < 0.05, **p < 0.01, ***p < 0.001, and ****p < 0.0001. [file PHY2-13-e70373-s002.jpg]

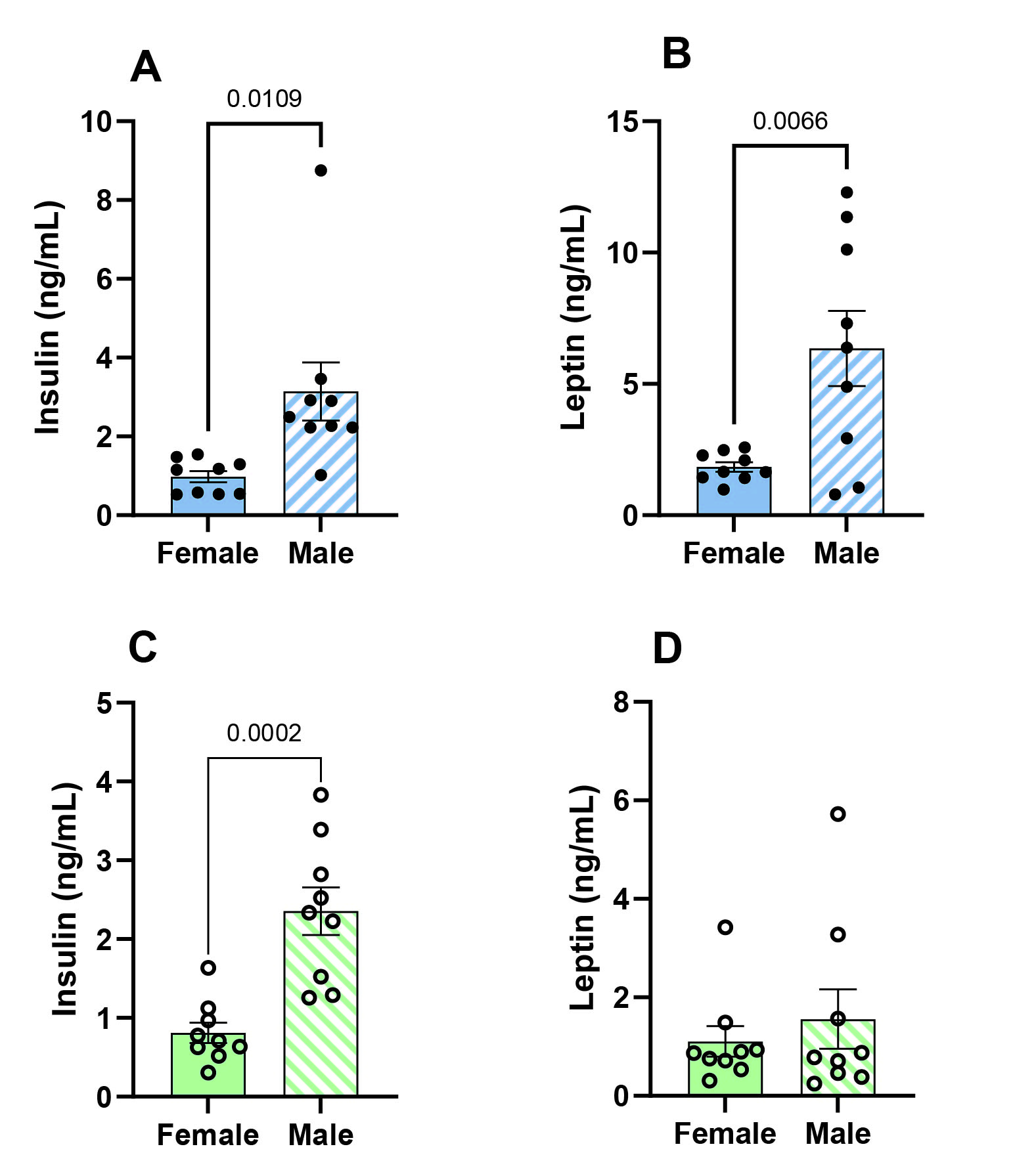

Supplement: Supplementary file 5 — Figure S5: Sexual Dimorphism in Insulin and Leptin Levels. Conventional male and female comparisons of insulin (a) and leptin (b). Germ‐free male and female comparisons of insulin (c) and leptin (d). Two‐way ANOVA with Sidak’s Multiple Comparisons Test (a, b) and unpaired t‐test (c‐f) with *p < 0.05, **p < 0.01, ***p < 0.001, and ****p < 0.0001. ROUT’s Test to Identify Outliers (a). [file PHY2-13-e70373-s005.jpg]
