# Supplementary material for: Microbiome affects mice metabolic homeostasis via differential regulation of gene expression in the brain and gut
Source: Physiol Rep. 2025 May 19;13(10):e70373. doi: 10.14814/phy2.70373 (PMC12087290; doi:10.14814/phy2.70373)
Supplement: Supplementary file 6 — Table S1: Primer sequences used for qPCR. Each primer is listed with the gene, primer name, and DNA sequence. Each primer set defines which primer is the forward or reverse primer. [file PHY2-13-e70373-s006.docx]

| **Gene** | **Primer Name** | **Primer Sequences** | **Forward or Reverse** |
| --- | --- | --- | --- |
| Beta-actin | Actb F | cagcttctttgcagctcctt | F |
|  | Actb R | cacgatggaggggaatacag | R |
| Agrp | Agrp-RT2-for LEO | cggcctgaaagctttgtcctc | F |
|  | Agrp-RT2-rev LEO | tgtagccagggcatgaggtg | R |
| Pomc | POMC RT-F2 | tagatgtgtggagctggtgc | F |
|  | POMC RT-R2 | ttttcagtcaggggctgttc | R |
| Socs3 | Socs3 F | cacctggactcctatgagaaagtg | F |
|  | Socs3 R | gagcatcatactgatccaggaact | R |
| Cck | mCCK F | gcactgctagcgcgatacatc | F |
|  | mCCK R | ccaggctctgcaggttcttaag | R |
| Gip | Ms Gip qF1 | aactgttggctaggggacac | F |
|  | Ms Gip qR1 | gaaagtcccctctgcgtacc | R |
| Gpr119 | Gpr119 mF2 | ccggagctcgatggctaag | F |
|  | Gpr119 mR2 | ggacaacctgcctttaccagt | R |
| Gpr120 | Gpr120 mF2 | gtctgccacctgctcttcta | F |
|  | Gpr120 mR2 | tgggccaatccaatgtgcaa | R |
| Gpr17 | Gpr17 qF2 | ggagcaccatctagaggaccctct | F |
|  | Gpr17 qR2 | ggctgcctccagaccgttcat | R |
| Gcg | Ms Gcg qF2 | ggcacattcaccagcgacta | F |
|  | Ms Gcg qR2 | gtcccttcagcatgcctctc | R |
| PYY | Ms PYY qF1 | cctgctcatcttgcttcgga | F |
|  | Ms PYY qR1 | actggtccaaaccttctggc | R |
| ApoB | ApoB qF1 | ctgtgggtctggattcgagc | F |
|  | ApoB qR1 | tagccttagaagccttgggc | R |
| Glut2 | Slc2a2 F1 | tcatgtcggtgggacttgtg | F |
|  | Slc2a2 R1 | cccaaggaagtccgcaatgta | R |
| Fatp4 | Fatp4 F1 | tgctcctgtacttggggtct | F |
|  | Fatp4 R1 | aatcagggctgtcttgtccg | R |
| Sis | Sis F1 | cccctagtcctggaaggtgt | F |
|  | Sis R1 | ctgacgcagcagtatagcca | R |
| Ffar3 | Ffar3 F1 | tgtccaatactctgcatctgtg | F |
|  | Ffar3 R1 | cacgaggaacaccaacaggta | R |
| Pck2 | Pck2 F1 | gaggctgagaacactgccat | F |
|  | Pck2 R1 | ccgtcttgctttctacccgt | R |

**Supplementary Table S1**. **Primer sequences used for qPCR**. Each primer is listed with the gene, primer name, and DNA sequence. Each primer set defines which primer is the forward or reverse primer.
